# Proteomic Advances in Milk and Dairy Products

**DOI:** 10.3390/molecules26133832

**Published:** 2021-06-23

**Authors:** Rubén Agregán, Noemí Echegaray, María López-Pedrouso, Radwan Kharabsheh, Daniel Franco, José M. Lorenzo

**Affiliations:** 1 Centro Tecnológico de la Carne de Galicia, Adva. Galicia n° 4, Parque Tecnológico de Galicia, San Cibrao das Viñas, 32900 Ourense, Spain; rubenagregan@ceteca.net (R.A.); noemiechegaray@ceteca.net (N.E.); danielfranco@ceteca.net (D.F.); 2 Department of Zoology, Genetics and Physical Anthropology, University of Santiago de Compostela, 15872 Santiago de Compostela, Spain; mariadolores.lopez@usc.es; 3 Business Administration, Faculty of Economics and Administrative Sciences, Applied Science University—Bahrain, Al Hidd 5055, Bahrain; radwan.kharabsheh@asu.edu.bh; 4 Área de Tecnología de los Alimentos, Facultad de Ciencias de Ourense, Universidad de Vigo, 32004 Ourense, Spain

**Keywords:** proteomics, proteome, proteins, milk, dairy products, electrophoresis, chromatography, mass spectrometry

## Abstract

Proteomics is a new area of study that in recent decades has provided great advances in the field of medicine. However, its enormous potential for the study of proteomes makes it also applicable to other areas of science. Milk is a highly heterogeneous and complex fluid, where there are numerous genetic variants and isoforms with post-translational modifications (PTMs). Due to the vast number of proteins and peptides existing in its matrix, proteomics is presented as a powerful tool for the characterization of milk samples and their products. The technology developed to date for the separation and characterization of the milk proteome, such as two-dimensional gel electrophoresis (2DE) technology and especially mass spectrometry (MS) have allowed an exhaustive characterization of the proteins and peptides present in milk and dairy products with enormous applications in the industry for the control of fundamental parameters, such as microbiological safety, the guarantee of authenticity, or the control of the transformations carried out, aimed to increase the quality of the final product.

## 1. Introduction

Proteomics is an area of knowledge that emerged in the field of biology as a consequence of the discovery of the human genome. The first time this expression was coined was in the mid-1990s as a need to create a specific vocabulary that would describe the work carried out at that time, which was the study of the proteome. This study includes all the proteins of a single cell, together with all the isoforms and modifications, the interactions between them, and their structure and higher-order complexes [1]. During the early days of proteomics, efforts were focused on the field of medicine, where great advances were made in the diagnosis of diseases. However, the potential of studying the proteome of a biological sample was spread to other fields such as animal production. In particular, great efforts have been carried out to understand the milk proteome, which is defined as complex with a wide variety of compounds, some of which have found to have biological activities of great interest. This study focusses on exploring genetic aspects, molecular pathways, and cellular functions involved in the production of milk, its quality, and safety [2].

Milk is a fluid that contains many low-abundance proteins (5% of the total protein fraction) and they are located in the whey or in the milk fat globule membrane (MGMF) [2]. To this inconvenience must be added the presence of numerous genetic variants and post-translational modifications (PTMs), such as glycosylation, phosphorylation, disulphide bond formation, and proteolysis, which generate a large number of protein variants from a single gene product [3], which adds even more difficulty to proteomic analysis. Therefore, there is not yet a single protocol that is capable of providing a complete analysis of the milk proteome. In the last few years, analytical chemistry has developed a series of methods that include fractionation and separation of the sample, its concentration and the detection of the proteins present with a resolution and sensitivities enough for a comprehensive determination of the proteome [4]. The potential represented by the use of proteomics in animals has favored the advancement of technology. Two-dimensional gel electrophoresis (2DE) is generally applied for the separation of proteins or protein subunits according to charge, followed by another separation by molecular mass in SDS-PAGE. After a staining process, the proteins displayed in the form of spots are characterized by mass spectrometry (MS) analysis. In a complementary manner, the sample can be subjected to a digestion process and the peptides obtained analyzed by MS after separation by chromatographic techniques [5]. These two methodologies are very widespread in the analysis of the milk proteome, with multiple operational variations that are discussed below. This review focused on advances in proteomics for dairy proteome analysis and the applicability of these advances in the safety, authenticity, and quality control of milk and dairy products.

## 2. Milk Proteins and Their Significance in Human Nutrition

Milk may be defined as the liquid excreted by the mammary glands of mammals in order to serve as food for the newborn. However, since ancient times, milk has been considered a very valuable resource by human beings due to its nutritional composition and easy of obtaining. This contextualized vision of milk as an exploitable product to satisfy a need has led to a change in meaning. Hence, from a technological point of view, a more consistent definition of milk would be the product obtained by milking of mammals, which are raised for the only purpose of producing milk [6]. Different species of animals are used for milk production. Currently, cow’s milk represents around 80% of world production, followed by buffalo (~15%), goat (~2.5%), sheep (~1%), and camel (~0.4%) [7]. On the other hand, the importance of milk in the human diet goes beyond its consumption as a raw product. The elaboration of fermented dairy products has become a thousand-year-old tradition, which includes a wide variety of foods with different textures, tastes, aromas, shapes, and sizes [8].

Milk is a nutritionally very complete food due to the balance of its different components. These components are not in a fixed proportion, but vary depending on factors, such as the animal species, breed, period of lactation, and diet [9]. For example, buffalo and sheep milks have a higher fat content (7.5% and 6.4%, respectively) than cow (3.3%) and goat milks (3.9%). These species together with camel are five of the most consumed milks in the world and their composition is presented in Table 1 [10]. Milk is a biologically designed product to provide the neonate with the necessary nutritional requirements, and in particular, the protein fraction is highly valued due to its high nutritional quality. The consumption of milk provides all the essential amino acids and other compounds, such as vitamin-binding and metal-binding proteins and various protein hormones [11]. The amino acid profile is similar to that of the egg, although limited in sulphur-containing amino acids. However, the variety and quantity of these molecules present in milk is enough to cover the daily needs defined in adults by the FAO/WHO/UNU [12]. Rafiq et al. [13] reported a good balance of essential amino acids in cow, sheep, goat, buffalo, and camel milks, especially branched-chain amino acids (leucine, isoleucine, and valine) regarding the daily needs mentioned above.

The unique properties of milk proteins and their technological impact by playing a prominent role in the production of dairy products has led to their exhaustive study and characterization [14,15]. This fraction of milk is composed of two types of proteins, caseins and whey proteins. The former is obtained after acid precipitation; at pH 4.6 the casein fraction is insoluble in milk. The remaining liquid is a solution of proteins (whey proteins), which also contains lactose, salts, vitamins, and other compounds at trace levels [14,16]. Regarding caseins, up to five types can be distinguish, α_s1_-casein, α_s2_-casein, β-casein, γ-casein, and κ-casein [17]. They represent around 80% of the proteins in cow’s milk and other commercial dairy species. They are in the form of colloidal particles of 50–600 nm in diameter, known as micelles. Important and particular features of milk, such as white color, stability to high temperatures or ethanol, and coagulation in the presence of rennet are due to this protein subfraction [18]. Casein micelles are structurally complex. They are formed by highly phosphorylated caseins, which interact and aggregate with calcium phosphate. The proportion of κ-casein (located on the surface) determines the size of the micelle, as well as many other properties, especially the stability against aggregation [19], a determining factor in the production of dairy products.

Whey proteins, also called serum proteins, comprise around 20% of total proteins and 0.7% of cow’s milk and, like caseins, they are subdivided into sub-fractions (β-lactoglobulin, α-lactoalbumin, and several minor proteins) [14]. The main protein in whey corresponds to β-lactoglobulin. It represents more than half of this protein fraction and, in turn, is subdivided into two genetic variants, differentiated by the substitution of a glycine residue (variant A) for another of aspartic acid (variant B). The β-lactoglobulin molecule is characterized by the absence of phosphorous and by the presence of two disulfide and one free sulfhydryl groups. Other abundant protein in whey is α-lactoalbumin, which account for approximately 13% of the total serum proteins. The molecule of this protein contains four disulfide linkages and, like β-lactoglobulin, does not have phosphorus. There are other proteins in much lower amounts in whey, such as inmunoglobulins, which comprise 2% of serum proteins, and are divided into four classes, IgG1, IgG2, IgA, and IgM, or bovine serum albumin (BSA) [20]. The major milk proteins of several highly consumed species, including human, are represented in Table 2.

Recently, the study of the milk proteins and related compounds has revealed multitude of beneficial properties for health. It is suggested that the consumption of milk and dairy products might help prevent and delay the appearance of certain types of cancer [21,22,23]. In this line, Kim et al. [24] reported that α-caseins, β-caseins, and β-lactoglobulin can protect cells from oxidative stress, inhibiting cellular senescence. These compounds might be used as supplements for the prevention of aging-associated diseases, especially in the prevention of skeletal muscle loss. 

Milk is a very nutritious medium, a natural source of fatty acids, essential amino acids, and the sugar lactose, which makes it an optimal medium for microbial growth. However, a number of compounds with antimicrobial activity, such as immunoglubulins, lactoferrin, lactoperoxidase, and lysozyme, can be found naturally in milk. In addition, antimicrobial activity has been also observed in peptides found in fermented dairy products [25]. These compounds help keep the raw product stable for longer and they might be used as nutraceuticals in novel food products.

**Table 2:** Major milk proteins of several highly consumed animal species.

Milk Compound (g/L of Milk)	Cow ^a^	Goat ^a^	Sheep ^b^	Buffalo ^c^	Camel ^d^
Crude protein	29.4	35.84	55.83	60.10	26.5
Casein	24	29.64	41.39	50.38	17.34
αs-casein	11.12	9.83	17.69	24.14	2.89
β-casein	12.88	19.80	13.95	18.45	12.78
k-casein			9.74	7.79	1.67
Whey protein	5.04	6.20	-	-	-
α-lactoalbumin	-	-	6.57	4.30	2.01
β-lactoglobulin	-	-	7.86	5.42	-

^a^ Ceballos et al. [26]; ^b^ Pelmus et al., [27]; ^c^ Bonfatti et al., [28]; ^d^ Omar et al., [29].

Overall, milk proteins, mainly those derived from whey, have been found to exhibit a wide variety of biological activities, acting as health promoters. Research in the field suggests that their use in the treatment of chronic diseases, such as obesity, hypertension, or type 2 diabetes could improve the prognosis of patients [30].

## 3. Milk Proteomics

### 3.1. Concept of Proteomics and Applied Strategies

Proteomics is a term that describes an area of knowledge framed within the field of biology formed by the combination of the words “protein” and “genomics”, that was first coined in mid-1990s, although the concept was proposed in 1979 by Anderson and Leigh when in the presentation entitled “Human Proteins Index Project” they hoped to unlock the genome by identifying the respective proteins using the recently developed technique of 2DE [31]. Wilkins et al. [32] was the first to stablish the concept of proteomics or proteome analysis, as the separation, identification, and quantification of the set of proteins expressed by a genome, cell, or tissue. Another definition could be that given by Anderson et al. [33], who determined the concept of proteomics as “the use of quantitative protein-level measurements of gene expression to characterize biological processes and decipher the mechanisms of gene expression control”. Basically, by the term of proteomics we refer to the set of proteins encoded by the genome, and by proteomics to the study of these proteins. However, this is a simplistic conception of the term, since proteomics not only includes all the proteins in a given cell, but also the set of all isoforms and modifications of the proteins, the interactions between them, and the description of their structures and higher-order complexes [1].

The characterization of proteomes is an arduous and complex task given the vast variability of the forms in which proteins can appear. Unfortunately, at present there are no methods available that allow the complete analysis of proteomes in a single and simple event. However, technologies developed in recent decades have made it possible to exponentially improve speed, accuracy, and level of detail [34].

#### 3.1.1. Separation Techniques

The proteome is a complex matrix that contains thousands of different protein molecules, the characterization of which requires analyzing each protein, its forms, and abundance within that matrix [34]. Hence, in order to correctly identify each protein, a previous separation step is necessary. The separation of all proteins contained in cells, tissues, and biofluids has been and continues to be a challenge for science, which for many years has tried to develop methods of fractionation, separation, concentration, and detection with sufficient resolution to separate large amounts of proteins, and with an adequate sensibility and dynamic range to detect those proteins present in low abundance [4].

**Figure 1:**
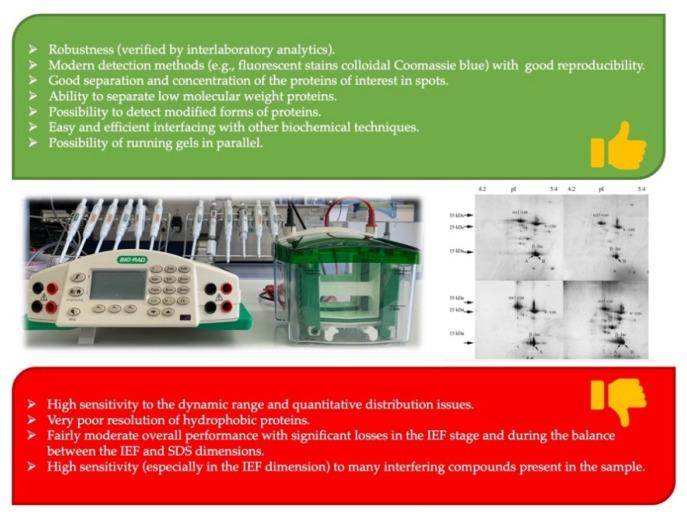
Advantages and disadvantages of two-dimensional gel electrophoresis (2DE) technology. Data from Rabilloud et al. [38]. Gel image from Zagorchev et al. [39].

To achieve protein separation, scientists have relied on electrophoretic and chromatographic technologies, separately and in combination, both offline and online [35]. Regarding the first of the aforementioned technologies, electrophoresis, one of the most popular and effective methods is the two-dimensional polyacrylamide gel electrophoresis (2D-PAGE) based on two-dimensional gel electrophoresis (2DE) technology (Figure 1). This method is based on the combination of two orthogonal separation techniques, isoelectric focusing (IEF) and sodium dodecyl sulphate (SDS) PAGE. First, with IEF technique, proteins are separated according to their different isoelectric point (pI) (first dimension). Then, proteins are separated again, but this time based on their electrophoretic mobility using SDS-PAGE (second dimension). Finally, the proteins are visualized and quantified by different procedures, including Coomassie, silver, or fluorescence stains [36]. The two-dimensional SDS-PAGE technique is usually not very effective in resolving proteins with similar molecular mass. Similarly, proteins with a similar pI are difficult to resolve using IEF gels, particularly when trying to analyze samples with high concentrations of individual proteins, such as milk. However, using a two-dimensional approach, these limitations are largely resolved, greatly improving resolution [3]. Despite the high strength showed by 2D-PAGE in the separation of proteomes, a significant number of disadvantages have been found, such as the low ability to detect proteins with low abundance [37], the scarce efficiency to separate polypeptide chains with molecular masses outside the 150-8 kDa range, or the difficulty of resolution of proteins with an extremely acidic (pI < 3) or basic (pI > 12) pH [3].

The utility and protein resolution capacity of 2D-PAGE has been reported in analysis of milk proteomes. Hsieh et al. [40] identified up to 15 proteins in cow’s milk, three α_s1_-caseins, three α_s2_-caseins, three β-caseins, three κ-caseins, two β-lactoglobulins, and one serum albumin, in a study that intended the proteomic analysis of the effects of chymosin on the coagulation of individual milk proteins. By using 2D-PAGE, D’Auria et al. [41] managed to detect the presence of four main protein spots, corresponding to albumin, caseins, β-lactoglobulin and α-lactalbumin, in milks from different animal species, included human. Up to eight and nine different proteins were separated and identified in human and cow’s milk, respectively.

Liquid phase separation is another separation method increasingly used that includes techniques, such as capillary electrophoresis (CE) or liquid chromatography (LC) [42,43,44,45,46]. The increase in its use in proteomic analysis is due to its numerous advantages over 2D-PAGE, such as its great sensibility, superior dynamic range, easy automatization, and speed. On the other hand, this method is very versatile, since it can work with different separation mechanisms (e.g., size exclusion, reverse phase, and ion exchange) and in this way, it can analyze basic or acid proteins and of any molecular mass. However, liquid phase separation suffers in term of resolution, being lower than using 2D-PAGE [47]. However, Omar et al. [29] highlighted the excellent resolution of the CE technique, greater than that of 2D-PAGE, in the separation of camel milk proteins. Using this technique, these authors managed to identify and quantify the major caseins (α-casein, β-casein, and κ-casein) and whey proteins (α-lactoalbumin, serum albumin, and lactoferrin).

#### 3.1.2. Characterization Techniques

Once the proteins have been selected, their identification or comprehensive characterization is carried out preferably using MS. Recent advances in this determination technology have allowed proteins to be characterized in a rapid and exhaustive manner [47], establishing itself as a powerful technology for protein characterization [48]. A mass spectrometer basically ionizes analytes using an ion source and provides information about the mass-charge ratio (*m*/*z*) of these and the number of ions at each value of m/z, using a mass analyzer and a detector, respectively. Moreover, in the case of peptides, a previous breakdown step is necessary through a process called dissociation, for which there are different techniques, such as collision-induced dissociation (CID), electron-capture dissociation (ECD), or electron-transfer dissociation (ETD) [49]. Electrospray Ionization (ESI) and Matrix Assisted Laser Desorption/Ionization (MALDI) MS techniques have greatly improved the biomolecule analysis, including proteins. The MALDI technique is frequently used for the characterization of simple proteomes. It has the advantage that it is partially resistant to interferences caused by buffers commonly used in protein analysis, such as phosphate, tris, or urea. In this way, the formation of singly charged ions is promoted, thus facilitating the interpretation of the generated mass spectra. These advantages have led to the use of MALDI-MS in many analytical areas. However, some important drawbacks persist, such as the variability of the signal intensities and the resolution between points of the same sample [50]. At this point, a delayed extraction technique could increase resolution, allowing peaks with a similar mass range to be differentiated. Cozzolino et al. [51] successfully applied this technology along with MALDI-MS, using a time-of-flight (TOF) detector as a rapid and easy strategy to identify possible adulteration in raw milk samples. The limited resolution of MALDI was partially resolved by the delayed extraction technique, which allowed to separate peaks with a mass range of up to 20-30 kDa, using the main whey proteins α-lactalbumin and β-lactoglobulin as molecular markers. The authors proposed this protocol for the detection of cow milk in ewe and water buffalo milks, which proved to be practical and fast enough, managing to analyze around 100 samples in less than an hour. In contrast, the ESI technique produces the generation of multiply charged ions. An important advantage of ESI is its easy in line coupling with high-performance separation techniques such the aforementioned CE and HPLC [52]. Vincent et al. [53] greatly optimized the separation of intact cow milk proteins by HPLC and their analysis by MS using ESI as ionization source. The quantification of proteins was successfully applied to Holstein-Friesian and Jersey milks to assess differences in proteins variant levels.

In recent years, the evolution in MS, with the appearance of new instruments, has led to the development of new ion activation techniques and reduced the detection limit. In addition, the latest evolutions of this technology have achieved to improve the understanding of structures of peptides and proteins [54]. However, the identification of these compounds remains particularly difficult since the data libraries do not always offer reliable results. Furthermore, negatives, false positives, and unassigned spectra can be produced [49]. Along with these drawbacks, it should be noted the unaffordable price for a fragile technology that requires continuous maintenance.

### 3.2. Advances in Proteomics in the Characterization of Milk and Dairy Products

During the 1980s, 2DE procedures were used extensively for the mapping of milk proteomes, including that of some dairy products. Thus, the protein patterns of different milks were obtained by using the aforementioned IEF and SDS-PAGE techniques, which has made it possible to compare their most abundant proteins. The quantification of protein expression in images enables this comparison among experiments, popularizing the use of 2DE gels [55]. Nevertheless, low abundant proteins remained undetectable. With the emergence of immobilized pH gradients (IPGs) this problem was partially solved, increasing the resolution for a better visualization of the protein profile [56]. In this manner, Conti et al. [57] were able to carry out the isolation and subsequent characterization of a genetic variant of bovine β-lactoglobulin. By means of a preparative HPLC gel filtration and preparative IEF-IPG they reached a highly purified protein.

Although the use of 2DE technology was a milestone in the early days of proteomics and provided great results in the past, the reality is that its use as the sole protein characterization technique allows only a part to be identified from the analytical sample. The combined use of 2DE technology with specific detection systems has increased sensitivity when characterizing protein profiles. The coupling of 2DE to MS was one of the most important advances in milk proteomics. Protein analysis using this combination of technologies in which identification is achieved by MS after proteins have been separated by 2DE and digested with trypsin represents an interesting methodology with a powerful application in the study of dairy products [58]. Up to 30 proteins were identified successfully through this strategy in the aqueous extract of Swiss-type of cheese during the ripening stage, including those proteins expressed by *Streptococcus thermophilus* ITG ST20 and *Lactobacillus helveticus* ITG LH1 microorganisms used as starters [59]. Similarly, Pourjoula et al. [60] characterized the proteome and peptidome of kashz, a typical Iranian dairy product made from sour milk, combining technologies such as SDS-PAGE and HPLC separation techniques coupled to MS. The results showed a protein profile similar to yogurt and exposure to in vitro plasmin hydrolysis reveled that it is actually a product resulting from acid coagulation in the absence of γ-caseins.

Milk proteome has been found to be highly heterogeneous due to the existence of numerous protein isoforms [61]. These modifications may arise from alternative mRNA splicing, single point mutations, and PTMs. These changes in protein structure often cause variations in the molecular mass and net charge [62]. Some of these isoforms, the so-called PTMs are very important when characterizing the proteome as they influence some structural or functional aspect of the protein involved. These modifications generally originate after protein synthesis on cellular ribosomes [63,64]. PTM is the second pathway after genetic polymorphism by which the milk proteome expands, notably increasing its complexity. Many proteins undergo a multitude of PTMs, including phosphorylation, acetylation, glycosylation, disulfide crosslinking, lipid conjugation, and proteolytic cleavages, critical for important functions of milk proteins, such as micellar stability [64], vital for making cheese. Many of these particular modifications have been found to be affected by different factors. Thus, Fang et al. [65] reported variations in the relative concentrations of α_s_-casein phosphorylation isoforms in cow milk depending of intrinsic and extrinsic factors. The use of LC-ESI MS for the proteome characterization showed that parity, lactation stage, and genetic variation among animals positive contributed to phenotypic variation in the relative concentrations of individual α_s_-casein phosphorylation isoforms and in the phosphorylation degree of α_s_-caseins. Variations in the phosphorylation degree of this kind of caseins play a major role in the milk industrial properties. When investigation the causes that differentiate good coagulation from poor coagulation, even its absence, Frederiksen et al. [66] found that the presence of a lower proportion of two less phosphorylated variants of α-casein (α_s1_-casein-8P and α_s2_-casein-11P) in Danish Holstein milk is related to worse coagulation compared to other milk samples were its presence was higher. Less significant appears to be the level of phosphorylation in κ-casein for milk coagulation. In a study carried out with the Holstein and Jersey cow breeds, six isoforms of κ-casein were identified that varied in the levels of phosphorylation and glycosylation (95 to 96% of the total κ-casein was phosphorylated, while 34 to 35% of the κ-casein isoforms were glycosylated). However, despite this, the authors of the study reported a very consistent κ-casein PTM patterns independent of milk coagulation ability [67].

These modifications in protein molecules have technological consequences for the dairy industry. Thus, protein functions such as micellar stabilization, among others, are governed by protein phosphorylation-dephosphorylation reactions. Advances in proteomics have improved the identification of these compounds. Through an approach using the connection of several mass analyzers (MS/MS), it has been possible to decipher proteins of the membrane of milk fat globules of different species [68]. Pisanu et al. [69] managed to identify 140 proteins belonging to this membrane in sheep’s milk using LC tandem mass spectrometry (LC-MS/MS). The connection of several mass analyzers in series allows more detailed information regarding the molecular structure of the compound. Equipment with ESI-MS or MALDI-MS are really effective for the determination of molecular ions and are sometimes even capable of providing valuable structural information. However, to obtain more detailed information regarding the molecular structure of the compound it is necessary to connect several serial mass analyzers. In this way, the first analyzer isolates the ions and then their fragmentation occurs. Subsequently, the final analyzer separates these fragments based on their m/z values [70]. Recent original research studies have shown the great utility of this evolution of MS. Milkovska-Stamenova and Hoffmann [71] identified 14 types of protein-derived advanced glycation end products (AGEs) produced during dairy processing and storage using nanoRP- ultraperformance liquid chromatography (UPLC)-ESI-MS/MS. These AGEs can alter protein functions, decrease nutritional value, and produce other potentially harmful health effects. For at least one identified AGE, 132 modified peptides were found, with AGE-amide being the main group of compounds. The identification of low abundance proteins in milk and dairy products has recently gained importance due to the proven beneficial health effects of a large quantity of peptides and proteins. Tacoma et al. [72] found a total of 935 low abundance proteins in the skimmed milks of Holstein and Jersey cows using LC-MS/MS. In the study, it was observed that a total of 43 of these proteins were expressed differentially between the two breeds. Similarly, Verma et al. [73] used nano-scale liquid chromatography tandem mass spectrometry (nLC-MS/MS) for the identification of 1307 functional proteins comprising casein and other low abundance proteins. The authors reported that the vast majority of these proteins were involved in binding function and catalytic and structural activities.

### 3.3. Benefits of Recent Proteomic Advances in Milk and Dairy Production

In the dairy industry, the protection of consumer health and the guarantee of a nutritionally similar milk to the original has become an essential task and a large part of its efforts lies in guaranteeing these quality parameters. The origin of the milk is fundamental, since for example, that from a sick cow with mastitis can present a high number of somatic cells, which leads to an altered concentration of caseins, salts, free fatty acids, or lactose. Thus, a poor-quality milk will affect the dairy products made from it. In order to identify this type of anomaly, it is necessary to analyze protein patterns, as well as PTMs and even the presence of allergens [74], in order to provide the pertinent information aimed at protecting the consumer.

#### 3.3.1. Ensuring the Safety of Milk and Dairy Products

**Figure 2:**
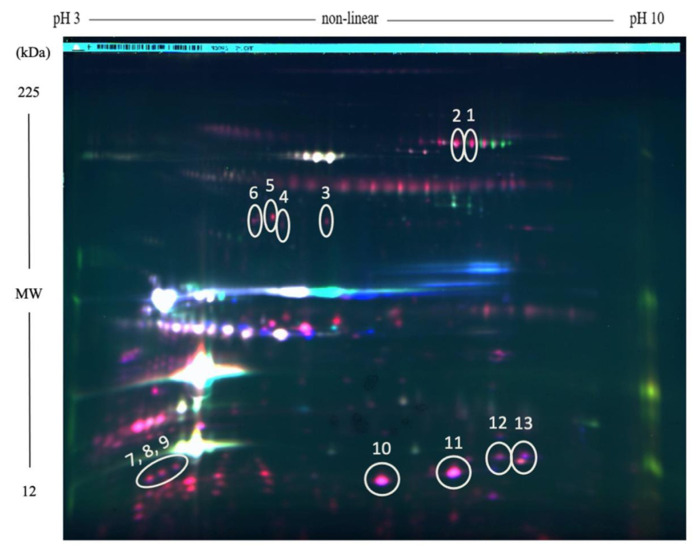
Proteins in mastitic milk (red spots) vs proteins from healthy milk (green spots) in two-dimensional differential gel electrophoresis (2D-DIGE) image. Proteins with similar levels in both groups are indicated by white spots [80].

Proteomics has been a revolutionary technology for the analysis of samples and the early detection of diseases that can affect the entire production chain in the dairy industry, such as mastitis. MALDI-TOF MS has helped to overcome this problem by improving the detection of bacteria in sample by identifying biomarkers that represent ribosomal proteins from different bacteria. This methodology has improved to microbiological methods where there is an inherent error in the sample preparation and conditions of the culture medium, which can now be solved [74]. With regard to the aforementioned mastitis disease, it usually causes the discard of numerous liters of milk in the farms if it is not prevented or controlled properly, in addition to representing an industrial problem if it goes unnoticed. It is defined as an inflammatory disease due to infection of the udder tissue, caused by numerous pathogenic microorganisms, but usually staphylococci, streptoccci, and enterobacteria are involved, with *Staphylococcus aureus* and *Streptococcus agalactiae* being the most contagious and responsible for the vast majority of infections [75]. When the disease occurs, milk is not adequate for transformation and changes in the nutritional profile are produced. Ogola et al. [76] observed an increase in the levels of non-casein fractions, sodium, chloride, and free fatty acids, while the casein content, lactose, caseins, potassium, and calcium decreased respect the normal levels present in the milk from a healthy cow. This disease is characterized by the presence of a high number of somatic cells that in turn are related to a variable proteome [77]. Therefore, proteomics represents an important advance in the field of early detection of this anomaly in milk. Pisanu et al. [78] found the increase of 119 proteins related to innate immune defense or structural functions in buffalo milk with mastitis after using SDS-PAGE and LC-MS/MS, which included vimentin, cathelicidins, histones, S100 and neutrophil granule proteins, haptoglobin, and lysozyme. In the same experiment, the authors also identified up to 33 lowering proteins that were found to be involved in lipid metabolism, including butyrophilin, xanthine dehydrogenase/oxidase, and lipid biosynthetic enzymes. In the same line, Abdelmegid et al. [79] used proteomics to find changes in the protein profiles in the whey of cows suffering from mastitis in order to identify biomarkers that help diagnose the disease. Using two-dimensional differential gel electrophoresis (2D-DIGE) coupled with LC-MS/MS, 28 highly abundant proteins were identified in milk affected by *S. aureus* (Figure 2). Nine of these proteins were involved in host defense functions, such as acute phase proteins, which play a role in the innate immunity and antimicrobial functions (e.g., serotransferrin, complement C3, fibrinogen γ-B chain, and cathepsin B), and proteins associated with the immune response to pathogens, such as polymeric immunoglobulin receptor-line protein, MHC class I antigen, and β-2-microglobulin.

Bacteria play a leading role in the transformation and maturation of dairy products, as long as they are selected and introduced in a controlled manner. However, when those so-called pathogens take control of the biochemical reactions, the situation takes on a worrying turn and if it is not corrected in time, it may cause a serious economic cost and a public health problem. An example of this problem occurs in what is perhaps the most universal dairy product, cheese. In most cases, cheese is considered a safe and stable food. However, there are a number of pathogenic microorganisms that can be present in dangerous quantities, such as *Listeria monocytogenes*, *S. aureus* and *Escherichia coli*, and represent a risk to consumer health [74]. In the last few years, several cases of outbreaks of these microorganisms related to cheese consumption have been reported [80,81,82,83]. In this field, proteomics techniques could be a useful tool in the detection and control of these microorganisms. Mendonça et al. [84] successfully applied MALDI-TOF MS/MS for the characterization of fructose 1,6-bisphosphate aldolase (FBA) and proposed it along with mAb-3F8 against this protein as molecules capable of specifically recognizing the genus *Listeria*. Western and dot blot assays further demonstrated that mAb-3F8 together with another antibody (anti-InlA mAb-2D12) could differentiate bacteria species from artificially contaminated cheese. In addition, localization studies indicated that FBA is present in every fraction of the *Listeria* cells, including the supernatant and the cell wall. However, the excessive protein content of cheese could be an impediment in the pathogen identification. Karasu-Yalcin et al. [85] reported a delay in the detection of *L. monocytogenes* in foods with a high protein content. One of the reasons for this difficulty in detection was the possible increase in interfering spectral peaks due to the diversity of components in the medium. Furthermore, the identification of the bacteria in white cheese did not provide reliable results. The same difficulty was found to identify *L. monocytogenes* in camembert cheese. Jadhav et al. [86] achieved a successful detection after inoculating 10 cfu/mL of the pathogen to the cheese and an incubation period of 30 h. More research must be carried out to tackle these problems and arrive at a reliable, direct, and rapid detection of disruptive microorganisms that allows the implementation of the proteomic technology in the industry.

Allergenic proteins are present in various widely consumed foods, such as wheat and soy or eggs and fish, and may represent a health problem for the consumer. The ingestion of these proteins generates an adverse immune response to those hypersensitive individuals, which can cause anaphylaxis, even death [87]. The consumption of cow milk is one of the most frequent food allergies. The proteins α-lactalbumin, β-lactoglobulin, and casein are usually those that often cause some problem of hypersensitivity, especially in infants. However, all cow milk proteins can be potential allergens, even those that are present at trace levels [88]. Correct labeling is essential to prevent consumer health problems. Therefore, the identification of allergens is essential, and the food industry is constantly developing to meet this market need. Detection and quantification of allergens by antibody-based assays are limited by matrix/processing effects and epitope masking. Traditionally, immunoassays such as the enzyme-linked immunosorbent assay (ELISA), which are based on the recognition of monoclonal or polyclonal antibodies have some disadvantages due to denaturation of food proteins and degradation of epitopes during the thermal process. MS represents an improvement in the proteomic analysis of new food allergens, since through targeted MS/MS techniques and data independent acquisition (DIA) methods it provides better selectivity, precision, and accuracy of quantification [89].

For allergen detection, an approach based on searching for specific peptides resulting from enzymatic digestion of protein extracts is often used. The selection of these peptides can be performed by preliminary in-silico selection of target proteins and peptides using advanced bioinformatics tools such as online databases for fasta sequences (Uniprot), searching tools for sequence alignment (BLAST), and free software for target proteomic method development such as Skyline, or by non-directed MS/MS analysis of samples with enzymatically digested allergenic ingredients present in artificially contaminated extracts and subsequent software-based protein/peptide identification. Once the specific marker peptides have been identified, the detection and quantification of allergens can be achieved using different MS technologies [90]. MALDI-TOF MS was used successfully for the identification of cow milk proteins and their isoforms reactive to IgE in children, detecting allergies to α_s1_-casein in 55% of the patients, 90% for α_s2_-casein, 15% for β-casein, 50% for κ-casein, 45% for β-lactoglobulin, 45% for BSA, 95% for the IgG-heavy chain and 50% for lactoferrin [91].

#### 3.3.2. Checking the Authenticity of Milk and Dairy Products

The adulteration of milk is another way through which its original characteristics can be modified or altered and its sale, as well as any product made from it, is a fraud to the consumer. Milk can be easily adulterated, and reasons for such deleterious activity may include disparate demand and supply, short shelf life of milk, reduced consumer purchasing power, and lack of adequate screening tests [92]. Proteomics offers a feasible solution for the detection of this type of fraudulent milk and its withdrawal from the market. The mixture of milk from different species is a common and easy type of adulteration that, in addition to being a fraud, may lead to health problems. Cow’s milk is a type of milk that, due to the presence of many allergens, is sometimes discarded for infant feeding and replaced by goat’s milk [93]. Fraudulent labeling could lead to a health problem. Di Girolamo et al. [94] were able to detect in a highly sensitive and robust way in comparison with other analytical tests for this purpose the adulteration of donkey and goat milks with cow’s milk up to a limit of 0.5% using MADLDI-TOF MS. The authors highlighted the ease of sample preparation, the speed, and reliability of the method to be used as routine analysis in the dairy industry.

The authenticity of a product such as cheese is very important, especially since there are numerous quality distinctions awarded based on its quality. Therefore, it is necessary to have a reliable method to detect possible fraud. The adulteration of cheese with milk cheaper than that registered on the label is one of the main problems of the dairy industry. The reference method for determining the presence of cow’s milk in sheep and/or goat cheeses for example is based on the different composition of the casein fraction of these species. Detection of γ2 and γ3 casein bands present in cow’s milk in an IEF gel has proven to be an effective method to verify possible fraud [95,96]. The combination of Urea-PAGE and reverse phase-high performance liquid chromatography (RP-HPLC) techniques together with a cluster analysis (CA) and principal component analysis (PCA) proved to be effective for determining protein profiles used as markers of authenticity of technological processes in Protected Denomination of Origin (PDO) cheeses [97]. Similarly, Rau et al. [98] described the MALDI-TOF MS technique as an easy, fast, and specific tool for the identification of the animal origin of milk in the production of feta and mozzarella cheeses. Using nanoLC-ESI-Ion Trap (IT)-MS/MS, Nardiello et al. [99] found that bovine species-specific peptides coming from β-lactoglobulin and αs1-casein could be used as markers for animal authentication of milk and detect alteration of goat milk at a level lower than 1%.

The addition of whey to raw milk to give a second life to this residue and increase profits is another type of fraud that can be detected if that milk is used to make cheese, since through the action of the enzyme chymosin the compound caseinomacropeptide (CMP) is released, a peptide that remains in serum and that can be used as a biomarker to detect possible adulteration using RP-HPLC or size exclusion liquid chromatography (SEC). However, some psychrotropic microorganisms, especially *Pseudomonas fluorescens*, can produce a peptide very similar to CMP with only one different amino acid residue, the detection of which detection is only possible by more selective techniques than those previously mentioned, such as LC-ESI MS, which demonstrated satisfactory precision to identify an adulteration in the milk destined to the manufacture of cheeses with a detection limit of 1 μg/mL and a quantification limit of 5 μg/mL [100]. Many efforts are being carried out to discover abnormalities in the protein profile related to the addition of other types of milk or derived products to that declared on the label of product in question. Certain deviations of the standard protein profile can be used to reveal possible quality alterations. In Table 3, interesting original research studies are presented in which proteomics is used for this purpose.

The addition of undeclared non-dairy protein is another illegal activity that affects the protein quality of the product. The low price of certain protein sources or their availability make their use attractive and their mixture with milk of animal origin can be a scam to the consumer. For example, evidence of adulteration of skimmed milk powder with vegetable proteins can be corroborated by using LC-MS and thus unequivocally discriminate the presence of non-animal protein. On the other hand, LC-MS/MS offers a more specific approach on the type of protein and its origin, being a powerful tool that would allow the identification of the added protein source [108]. 

#### 3.3.3. Industrial Operations of Milk and Dairy Products Affecting Quality

**Figure 3:**
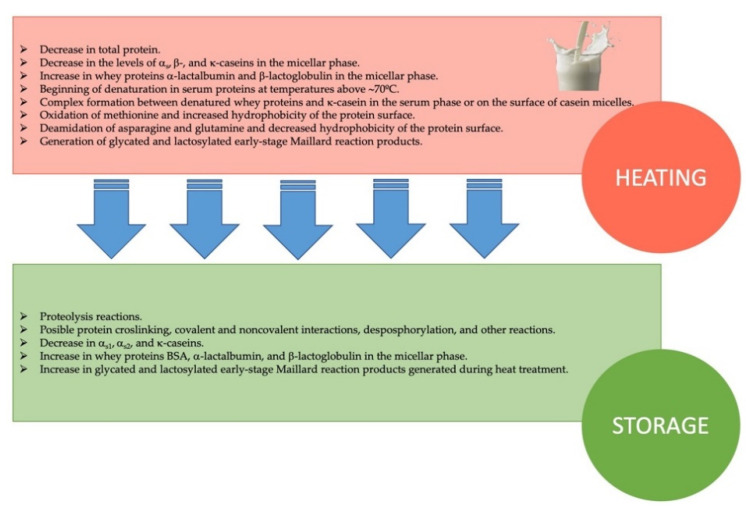
Possible modifications of milk proteins during heat treatment and subsequent storage. Data from Liu et al. [117].

In industry, milk is treated and/or transformed to give rise to different dairy products. During the different operations, changes in the protein profile may occur above what is acceptable, modifying the sensory characteristics and/or decreasing the nutritional quality. Therefore, the control of these parameters is vital to guarantee a product in optimal conditions to the consumer. Milk is a food that, due to its composition, rich in amino acids, fatty acids, and lactose, is a perfect medium for the development of microorganisms. When the milk is excreted by the mammary gland of the animal, it is considered a sterile product unless the animal suffers from an infection. From this moment, the milk is colonized by a wide microbiota, belonging to the skin of the teat and the epithelial lining of the teat canal, but there are also microorganisms that can migrate from the milking equipment, the feeding place of the animals, or the bedding material [109]. To guarantee the health of the milk and increase its shelf life, it is imperative to carry out a heat treatment in conditions to reduce the microbial load. Some of these treatments such as pasteurization and sterilization have been shown to be effective in achieving this goal. However, a heating process may produce a decrease in the food quality by affecting the color, flavor, or nutritional value [110]. It has been reported that heat could cause chemical modifications in certain heat-sensitive milk proteins, such as glycation, oxidation, denaturation, and aggregation, affecting their bioavailability and functionality [111]. Figure 3 shows probable protein modifications that occurred during milk heating. During the processing of some dairy products, such as powdered milk and milk protein concentrates, a strong Maillard reaction may occur and cause browning and product formation through protein crosslinking. In this field, proteomics can be used to detect changes in key milk proteins that provide valuable information to identify an altered product that is far from the original. Alpha-lactalbumin and β-lactoglobulin are susceptible to react with lactose during thermal treatment, losing functional properties and reducing the bioavailability of important amino acids. More intense glycation reactions were reported in lactose-free milk than in normal milk due to the higher reactivity shown by monosaccharides, such as glucose and galactose. The absence of the disaccharide lactose seems to promote the reaction of the whey proteins α-lactalbumin and β-lactoglobulin with the monosaccharides, forming adducts and damaging the milk integrity [112]. Other chemical changes in UHT milk and powdered milk have also been studied by proteomics, with positive and applicable results for the identification of dairy products with excessive heat treatment. In this way, it is possible to check and obtain a report of the thermal history of processed dairy products [113]. Ebner et al. [114] found 16 peptides present in drinking milks that could serve as indicators of abnormal heat treatment. The relative amount of these peptides was found to change as heat load increases. Especially sensitive to temperature variations was the peptide β-casein 196–209 (*m/z* 1460.9 Da), showing to be useful as a marker for monitoring milk processing conditions. Liu et al. [111] used proteomics to identify milk degraded by excessive application of heat. After submitting a cow’s milk to 85 °C for 5 min, they found a high degradation of the serum proteins lactoferrin, immunoglobulin, and lactoperoxidase by means of an analysis with LC-MS/MS compared to when non-thermal treatments such as ultraviolet-C and thermo-ultrasonication were applied, remaining practically intact. In addition, complement proteins, xanthine dehydrogenase/oxidase, and fatty acid-binding protein significantly decreased their concentration. In another study, five new peptides possibly associated with the casein fraction in cow’s milk were identified after being subjected to heat treatment. Subsequently, during storage, an increase in the concentration of a peptide was observed, which was identified by MALDI-TOF MS as the N-terminus of α_s1_-casein [115]. Indeed, during milk storage, a series of alterations may occur that affect milk quality (Figure 3). The proteolytic activity of naturally present enzymes might cause modifications in the peptidome as suggested by model experiments and, hence, the peptide profile again could be suitable to evaluate the status of the product both during storage and during processing [114]. During storage of commercial UHT milk, it was found that up to 22 peptides increased significantly due to proteolytic activity attributed to different mechanisms (e.g., endogenous proteases and microbial proteases). Ten peptides were selected as potential markers to measure the quality of milk, highlighting the peptide β-casein 196-206 (*m/z* 1668.9) as the most suitable to differenciate suitable UHT milk from that not suitable for consumption [116].

Cheese maturation is a very important process that affects the consistency and appearance of the product and, in addition, provides characteristic flavors and aromas through complex biochemical reactions. During this transformation, a number of components of milk are affected, including the protein fraction, especially caseins. The starter culture is responsible for glycolysis, proteolysis, and lipolysis reactions in the cheese that will cause a slow transformation of the product. During this transformation stage, there is a slow breakdown of proteins to produce polypeptides, peptides, peptones, and free amino acids, which will later be transformed into other compounds.

Proteomics can play a prominent role during ripening to understand biochemical changes in the cheese proteome and detect specific ripening markers at different time points and be used in evaluating both the authenticity and quality of the product [74]. The use of SDS-PAGE and Urea-PAGE revealed an anomalous hydrolysis during the ripening of a mozzarella cheese made with buffalo milk. The reached fragments, β- and α_s1_-caseins, identified by HPLC-ESI MS, indicated the use of stored milk or stored and pressed curd, or the reuse of unsold mozzarella cheese, to manufacture mozzarella [118]. Each cheese proteome is unique and can be used as a fingerprint that characterizes the product and differentiates it from the rest. In this line, Silva et al. [119] worked on the characterization of the proteome of Coalho cheese, a Brazilian artisan cheese, confirming 57 to 72 peptides by MS, of which 32 were successfully identified (11 from α_S1_-casein, three from α_S2_-casein, 15 from β-casein and three from κ-casein). The peptide profile might be used as an exclusive biomarker for this type of cheese and serve to guarantee the quality of the final product. SDS-PAGE together with MALDI-TOF MS is a suitable method to separate and identify non-hydrolyzed proteins during cheese ripening, such as serum proteins (α-lactoalbumin, β-lactoglobulin, and serum albumin). However, the limitation of SDS-PAGE technique when serum proteins predominate could be solved by RP-HPLC using MS and Edman degradation. For the detection of minority peptides, quadrupole (Q)TOF-MS technology is a good option, which in conjugation with N-terminal sequencing and LC-MS is capable of providing a more complete peptidome in aqueous cheese extracts [120].

#### 3.3.4. Future Prospects of Proteomics for Food Quality Control in Industry

The vast amount of information collected on proteomics during the last decades, developed technologies and available model systems provide a spectrum of mechanisms to be used in food technology [121]. Therefore, it constitutes an important tool for the development of quality control methodologies in production processes and for food safety [122]. Knowledge of the final protein profile of a food, such as milk or derivatives, is essential to guarantee its safety and suitability as a unique product characteristic of a brand, and proteomics has sufficient potential to ensure these quality parameters due to its ability to establish dynamic and reliable analytic methods that allow a dynamic analysis of food products throughout the chain. In fact, currently, proteomics is already capable of providing fast methods to study the proteome of complex foods, including raw materials and matrices [123] such as milk. Its applicability in quality control in the food industry must be supported by collaboration between the business entity and the scientific community for the development and optimization of methods and their implementation on an industrial scale. However, this implementation undoubtedly requires a strong economic investment that not all entrepreneurs are capable or willing to assume, since MS equipment, currently indispensable in proteomics, is not a cheap technology, it is fragile and also requires of constant maintenance. On the other hand, as it is a recent technology, the room for improvement is wide and innovation in this field is constant. Therefore, it is expected that the methods that involve expensive proteomic technology will become increasingly accessible and in the medium term can be considered to be incorporated into industrial protocols.

## 4. Conclusions

The proteome of biological samples has been studied for several decades, and many advances have been carried out to achieve a complete understanding of the entire network of molecules that compose it. The milk proteome is certainly complex, with up to 5% low-abundance proteins that are difficult to detect. At first, the 2DE separation techniques represented a technological revolution that allowed the quantification of proteins in a milky matrix. However, only a part of that matrix could be successfully identified. The combination of 2DE technology with MS has increased the sensitivity to characterize protein profiles of samples and represented an important advance in the study of the milk proteome. Recent advances in MS, such as ESI and MALDI techniques have allowed a faster and more exhaustive proteome characterization. All of this proteomic development has provided new methods for monitoring the safety, authenticity, and quality of milk and dairy products. Microbiological problems such as milk from cows with mastitis or contaminated milk and milks products may be detected early through the tools provided by proteomics. In the same way, proteomics has enough potential to detect fraud in industry, such as the use of undeclared milk on the label of a product or that of a non-dairy protein are. On the other hand, proteomics is also capable of monitoring or control transformations in industry. Thus, an excessive heat treatment that generates protein degradation products and/or the study of the characteristic protein profile of a cheese can be used as a guarantee of quality towards the consumer.

## Figures and Tables

**Table 1:** Nutritional composition of some of the most consumed milks in the world.

	Composition (g/100 g)				
	Cow	Buffalo	Goat	Sheep	Camel
Water	87.8	83.2	87.7	82.1	84.8
Protein	3.3	4	3.4	5.6	3.9
Fat	3.3	7.5	3.9	6.4	5
Lactose	4.7	4.4	4.4	5.1	4.2
Ash	0.7	0.8	0.8	0.9	0.9

Data from Muehlhoff et al. [10].

**Table 3:** Modified milk proteins after adulteration (markers) found in original research studies.

Adulteration	Protein Marker	Technology Used	Detection Limit	Reference
Ewe and water buffalo milks with cow milk	α-lactalbumin	MALDI-TOF MS	<5%	Cozzolino et al. [51]
	β-lactoglobulin			
Fresh cow milk with powdered milk	α-lactalbumin		_	
	β-lactoglobulin			
Goat milk with cow milk	β-lactoglobulin	ESI-MS	5%	Chen et al. [101]
Water buffalo milk and mozzarella cheese with cow milk	β-lactoglobulin	ESI-MS	_	Czerwenka et al. [102]
Sheep and goat milks with cow milk	α_s1_-casein	MALDI-TOF MS	5%	Calvano et al. [103]
	α_s2_-casein			
	β-casein			
	κ-casein			
	β-lactoglobulin			
Donkey milk with bovine or caprine milk	α-lactalbumin	MALDI-TOF MS	0.5-2%	Cunsolo et al. [104]
	β-lactoglobulin			
Mozzarella cheese made with bovine milk	β-casein	ESI-Q-TOF MS/MS	_	Russo et al. [105]
Ricotta cheese made with bovine milk	β-lactoglobulin	MALDI-TOF MS	5%	Russo et al. [106]
Fresh cow milk with powdered cow milk	α_s1_-casein	MALDI-TOF MS	<1%	Calvano et al. [107]
	α_s2_-casein			
	β-casein			
	κ-casein			
	α-lactalbumin			
	β-lactoglobulin			

## Data Availability

Not applicable.
